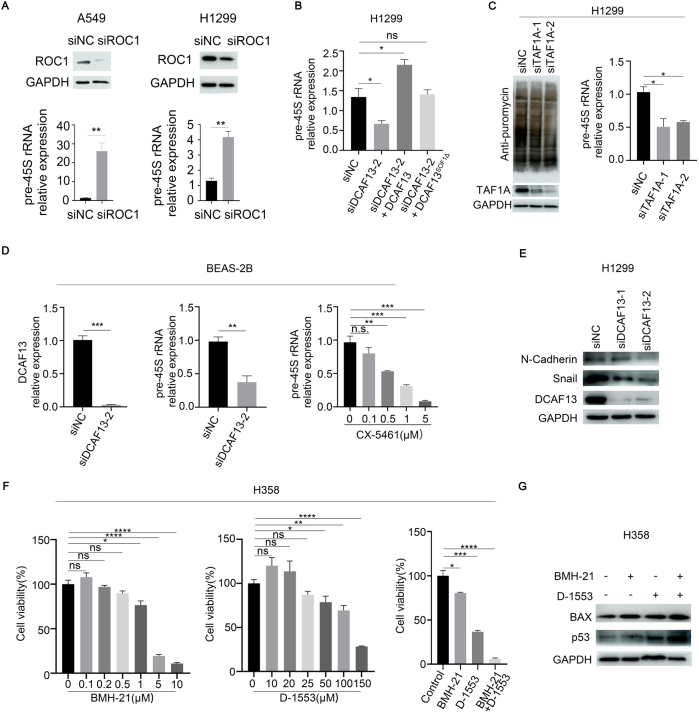# Nucleolar protein DCAF13 promotes non–small cell lung cancer cell proliferation *via* facilitating rDNA transcription and ribosome biogenesis

**DOI:** 10.1016/j.jbc.2025.110656

**Published:** 2025-09-01

**Authors:** Xiao-Min Wang, Meng-Dan Lv, Zhong-Jie Wu, Xiao-Hui Wu, Gu-Yuan Sun, Zi-Hao Wu, Xin-Kai Zou, Yu-Tao Wu, Le-Tian Liu, Le-Yi Yin, Yi Zhang, En-Hui Yang, Yong-Xia Zheng, Long Xu, Guo-Xin Hou, Yu Wang, Ya-Ling Zhang, Zi-Wei Hu, Sheng-Bing Liu, Lei Ao, Chun-Wei Xu, Michal Heger, Shu-Qun Cheng, Wei-Wei Pan

**Affiliations:** 1 Department of Basic Medicine, College of Medicine, Jiaxing University, Jiaxing, Zhejiang, P. R. China; 2 Department of Cardiothoracic Surgery, Affiliated Hospital of Jiaxing University, Jiaxing, Zhejiang, P. R. China; 3 College of Life Sciences and Medicine, Zhejiang Sci-Tech University, Hangzhou, Zhejiang, P. R. China; 4 Alberta Institute, Wenzhou Medical University, Wenzhou, Zhejiang, P. R. China; 5 Institute of Basic Medicine and Cancer (IBMC), Chinese Academy of Sciences, Hangzhou, Zhejiang, P. R. China; 6 Jiaxing Key Laboratory for Photonanomedicine and Experimental Therapeutics, Department of Pharmaceutics, College of Medicine, Jiaxing University, Jiaxing, Zhejiang, P. R. China; 7 Department of Hepatic Surgery VI, Eastern Hepatobiliary Surgery Hospital, Second Military Medical University, Shanghai, P. R. China; 8 Provincial Key Laboratory of Multimodal Perceiving and Intelligent Systems, Jiaxing University, Jiaxing, P. R. China; 9 Engineering Research Center of Intelligent Human Health Situation Awareness of Zhejiang Province, Jiaxing University, Jiaxing, P. R. China

**Keywords:** lung cancer, oncogene, cancer biology, ribosome biogenesis, transcription regulation, DCAF13, rDNA

## Abstract

The nucleolus is an important nonmembranous organelle within the nucleus and is responsible for ribosome biogenesis. This process is frequently upregulated in tumors to support elevated protein synthesis and sustain tumor growth. The transcription of ribosomal DNA (rDNA) into pre-45S rRNA, a rate-limiting step in ribosomal biogenesis, has been reported to be enhanced to drive the progression of non—small cell lung cancer (NSCLC). However, the mechanism that upregulating rDNA transcription in NSCLC remains unclear. Here, we show that rDNA transcript levels were increased in NSCLC tumor tissues. Reduction of rDNA transcription by CX-5461, an RNA polymerase I inhibitor, significantly inhibited NSCLC cell proliferation. We found that the expression level of DDB1- and CUL4-associated factor 13 (DCAF13), a nucleolar protein, positively correlated with that of pre-45S rRNA. DCAF13 knockdown impairs rDNA transcription, ribosome biogenesis, and protein synthesis, indicating that DCAF13 participates in rDNA transcription. Mechanistically, DCAF13 interacts directly with TAF1A, a component of the RNA polymerase I preinitiation complex. This interaction is necessary for preinitiation complex assembly. In addition, DCAF13 is highly expressed in NSCLC, and its expression is negatively correlated with the overall survival in NSCLC patients. DCAF13 knockout inhibited NSCLC cell proliferation, colony formation, and migration, indicating that DCAF13 is a poor prognosis indicator of NSCLC. In summary, we found that DCAF13 promotes NSCLC cell proliferation by coordinating with TAF1A to regulate rDNA transcription and maintain high ribosome biogenesis.

Lung cancer is one of the most common and the deadliest malignant tumors in the world. According to a statistical report from the International Agency for Research on Cancer, lung cancer affected approximately 2.2 million individuals in 2020, with 1.796 million deaths (1, 2). Non–small cell lung cancer (NSCLC) accounts for more than 80% of all lung cancer cases and can be divided into three major subtypes: lung adenocarcinoma (LUAD), lung squamous cell carcinoma (LUSC), and large-cell carcinoma (3, 4). Although molecular targeted therapy, immunotherapy, chemotherapy, and antiangiogenesis have achieved remarkable outcomes in the management of patients with NSCLC, the 5-year survival rate is still <15% (5). Therefore, there is an urgent need to better understand the molecular mechanisms that drive NSCLC development and to identify new therapeutic targets.

Pathologists have long observed that malignant cancer cells have altered nuclear morphologies such as increased nucleolar size or irregularly shaped nucleoli, which serve as hallmarks of malignant cells and underscore the connection between malignancy and the nucleolus (6, 7). Ribosome biogenesis occurs within the nucleolus through key steps including ribosomal DNA (rDNA) transcription, pre-ribosomal RNA (pre-rRNA) cleavage and processing, and ribosomal subunit assembly (8, 9). Ribosomes are translation machines for protein synthesis, mainly composed of rRNAs and a large number of ribosomal proteins. In eukaryotic cells, the ribosome consists of a small 40S subunit and a large 60S subunit. To support the vigorous metabolism and robust growth of tumors, abnormal increases in ribosome biogenesis often occur in tumors to maintain efficient protein synthesis for their growth (10). Accumulating evidence has shown an association between altered ribosomal biogenesis and multiple cancer types, including lung cancer (10, 11, 12). Thus, studying the molecular mechanisms underlying increased ribosomal biogenesis in lung cancer is of great significance.

The transcription of rDNA by RNA polymerase I into pre-45S rRNA is the rate-limiting step in ribosome biogenesis (13). Increased rDNA transcription is directly linked to elevated protein synthesis, which promotes cell growth. Notably, upregulation of rDNA transcription is associated with an increased risk of cancer development (14, 15, 16). For example, tumor drivers, such as c-Myc (17) and PI3K (18), drive rDNA transcription, increase ribosome production, and promote malignant transformation and cancer progression. In contrast, tumor suppressors, such as TP53 (19), PTEN (20), and Rb (21) inhibit rDNA transcription. In addition, Tsoi *et al.* found that pre-45S rRNA levels were significantly elevated in primary colorectal cancer tumor tissues compared with those in nontumor colon tissues and were associated with tumor, nodes, and metastasis (TNM) stages (22). In breast cancer, enhanced tumor aggressivity is associated with increased pre-45S rRNA synthesis (23). Specifically, oncogenes such as ECT2 and LINC01116 promote LUAD tumor initiation and growth by enhancing pre-45S rRNA synthesis and ribosome biogenesis (24, 25). In addition, Justilien *et al.* found that pre-45S rRNA expression was significantly higher in lung squamous cell carcinoma tumor tissues than in normal lung tissues (26). These results indicate a direct relationship between enhanced rDNA transcription and cancer progression. However, the mechanism linking rDNA transcription and ribosome biogenesis with NSCLC remains unclear.

DCAF13 (DDB1-CUL4-associated factor 13) was initially identified as a substrate-recognition protein in the CRL4 E3 ubiquitin ligase complex (24). DCAF13 has been demonstrated to participate in multiple biological processes, including early embryonic development (24), oogenesis and follicle growth (25), T-cell immunity (26), and tumor proliferation and metastasis, by mediating the ubiquitination of targeted substrates (27, 28, 29). Furthermore, a pan-cancer analysis of multiple databases indicated that *DCAF13* is highly expressed in LUAD, and its high expression is negatively correlated with the survival of patients with LUAD (30, 31). Mechanistically, CRL4-DCAF13 targets the tumor suppressor—P53—for polyubiquitination and degradation, thereby promoting LUAD progression (32). This indicates that *DCAF13* serves as an oncogenic gene involved in NSCLC progression. Notably, in addition to serving as a substrate adaptor of CRL4 ubiquitin ligase, DCAF13 acts as a ribosome biogenesis regulator. Zhang *et al.* revealed that DCAF13 is involved in the cleavage and processing of 18S rRNA in growing oocytes (25). Wada *et al.* found that WDSOF1 (also known as the DCAF13 protein) in the nuclear matrix of HeLa cells is a component of the ribosomal small-subunit processing body (33). Jansen *et al.* found that SOF1 (the yeast homolog of DCAF13) participates in rRNA precursor processing and 18S rRNA (34). However, whether DCAF13 promotes NSCLC progression by regulating ribosomal biogenesis remains unclear.

In the present study, we found that rDNA transcription levels were elevated in NSCLC tissues. The rDNA transcription inhibitor, CX-5461, significantly suppressed NSCLC cell proliferation by downregulating rDNA transcription. We observed that DCAF13 was localized to the nucleoli of NSCLC cells. Silencing *DCAF13* hampered rDNA transcription, ribosome biogenesis, and protein synthesis in NSCLC, indicating that DCAF13 participates in rDNA transcription. Mechanistically, DCAF13 interacted with TAF1A, a component of the RNA polymerase I (Pol I) transcriptional machinery, and the interaction between DCAF13 and TAF1A is necessary for rDNA transcription. In addition, we found that high expression of DCAF13 in NSCLC tumor tissues correlated with poor prognosis in patients with NSCLC. *DCAF13* knockout significantly inhibited the proliferation, colony formation, and migration of NSCLC cells. Thus, our findings suggest that *DCAF13* promotes NSCLC progression by upregulating rDNA transcription and ribosome biogenesis.

## Results

### Pre-45S rRNA is highly expressed in NSCLC

**Figure 1:**
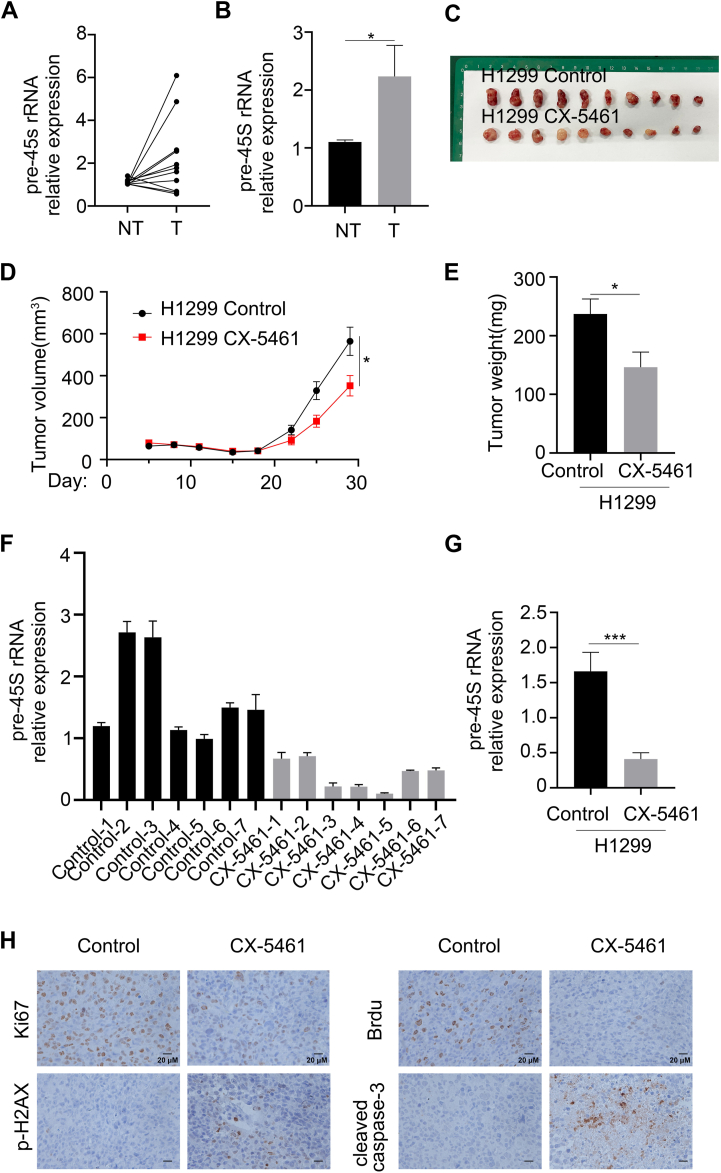
**Upregulation of pre-45S rRNA in NSCLC patients.***A*, Pre-45S rRNA was upregulated in tumor tissues obtained from NSCLC patients in the affiliated hospital of Jiaxing university. *B*, the average relative expression of pre-45S rRNA in paired adjacent normal and tumor tissues was compared. ∗*p* < 0.05, unpaired *t* test. *C*, H1299 cells were injected subcutaneously into both flanks of nude mice (5 × 10^6^ cells per mouse, n = 6 per group). The mice were divided into two groups, and each fed with water (Control) and CX-5461 (CX-5461) every 2 days. The dosage of CX-5461 is 50 mg/kg. The tumor-bearing mice were sacrificed on day 26. Representative photographs of excised tumors are shown. *D*, the volume of subcutaneous tumors in the control group and CX-5461 treatment group was monitored every 2 days. ∗*p* < 0.05, unpaired *t* test. *E*, tumor weight was determined at the end of the experiments. ∗*p* < 0.05, unpaired *t* test. *F*, RNA was extracted from the tumors in control group and CX-5461 group. The inhibition efficiency of pre-45S rRNA in the tumors was determined by qPCR. Data are mean ± SEM. *G*, the average relative expression of pre-45S rRNA in control and CX-5461 group tumor tissues was compared. ∗*p* < 0.05, unpaired *t* test. *H*, immunohistochemical analysis of Ki67, Brdu, p-H2AX and cleaved Caspase-3 in control and CX-5461 group tumor tissues. NSCLC, non–small cell lung cancer; qPCR, quantitative real-time PCR.

We first compared the expression level of pre-45S rRNA in paired tumor and adjacent normal tissues in patients with NSCLC from the Affiliated Hospital of Jiaxing University. We found that pre-45S rRNA was highly expressed in NSCLC tumor tissues compared with adjacent nontumor tissues (Fig. 1, *A* and *B*), suggesting that NSCLC tumors exhibited higher rDNA transcription levels. CX-5461 is a specific RNA polymerase I inhibitor that suppresses rDNA transcription and reduces ribosomal biogenesis. To verify whether rDNA transcription is necessary for the proliferation of NSCLC cells, we subcutaneously inoculated NSCLC cells (H1299) into nude mice and tested whether CX-5461 could inhibit tumor cell growth *in vivo*. As shown in Figure 1, *C*–*E*, the tumor volume and weight in the CX-5461 treatment group were significantly reduced compared with those in the control group, indicating that CX-5461 significantly suppressed H1299 cell growth. Quantitative PCR showed that the expression level of pre-45S rRNA in the CX-5461 treatment group was significantly lower than that in the control group (Fig. 1, *F* and *G*). Immunohistochemical results showed that the expression of proliferating marker, Ki67 and Brdu were significantly decreased in the CX-5461 treatment group, while the expression of DNA damage marker p-H2AX and apoptotic protein cleaved caspase-3 were increased (Fig. 1*H*). These results indicated that CX-5461 inhibited the proliferation of NSCLC cells and induced apoptosis of NSCLC cells by decreasing the expression of pre-45S rRNA. These results suggest that rDNA transcription plays an important role in NSCLC cell proliferation.

### Inhibition of pre-45S rRNA expression decreased the growth of NSCLC cells

**Figure 2:**
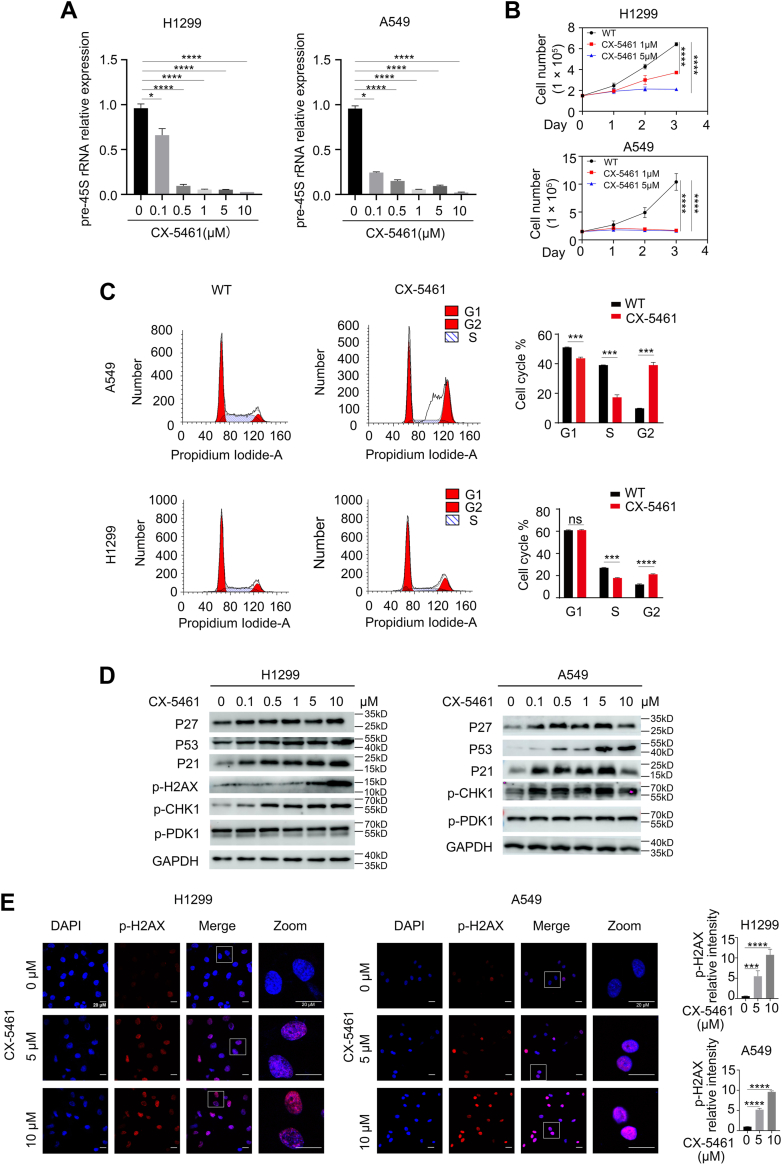
**pre-45S rRNA was essential for supporting the growth of non–small cell lung cancer cells.***A*, qPCR results for the changes in the expression levels of pre-45S rRNA in non–small cell lung cancer cell lines (H1299 and A549) treated with increased concentrations of CX-5461 for 24 h. ∗*p* < 0.05, ∗∗∗∗*p* < 0.0001, unpaired *t* test. *B*, growth curve assay for H1299 and A549 cells after treatment with 1 μM or 5 μM CX-5461. Cells (150,000) were plated in six-well culture dishes and cells were counted on days 1, 2, and 3. The data (N = 3 per cell line) are plotted as mean ± SEM. ∗∗∗∗*p* < 0.0001, unpaired *t* test. *C*, cell cycle phase determined by flow cytometry. H1299 and A549 cells were cultured overnight, treated with or without CX-5461 (5 μM), and then subjected to PI staining and FACS analysis. The percentages of cells in the G1, S, and G2 phases are indicated. ∗∗∗*p* < 0.001, ∗∗∗∗*p* < 0.0001, unpaired *t* test. *D*, western blot results showing the changes in the levels of the indicated proteins in H1299 and A549 cells treated with increased concentrations of CX-5461 for 24 h. *E*, immunofluorescent staining results showing increased pH2AX levels at 24 h after treatment with 5 μM or 10 μM CX-5461. The pH2AX relative intensity is the total pH2AX intensity divided by the DAPI area calculated by Image J software. ∗∗∗*p* < 0.001, ∗∗∗∗*p* < 0.0001, unpaired *t* test. DAPI, 4′,6-diamidino-2-phenylindole; PI, propidium iodide; FACS, fluorescence-activated cell sorting.

We further investigated whether the inhibition of pre-45S rRNA expression by CX-5461 suppresses the growth of the NSCLC cell lines H1299 and A549. We found that CX-5461 treatment significantly inhibited the expression of pre-45S rRNA in both H1299 and A549 cells at a low concentration 0.1 μM (Fig. 2*A*). As the concentration of CX-5461 increased to 0.5 μM, pre-45S rRNA expression decreased to a very low level. Moreover, CX-5461 treatment effectively inhibited NSCLC cell proliferation, indicating that suppression of pre-45S rRNA expression reduced the growth of NSCLC cells (Fig. 2*B*). To test whether CX-5461 treatment decreased NSCLC cell proliferation by altering the cell cycle, we investigated the effect of CX-5461 on cell cycle distribution. Fluorescence-activated cell sorting analysis revealed that CX-5461 treatment resulted in the arrest of NSCLC cells at the G2 phase of the cell cycle (Fig. 2*C*). It is well known that overexpression of cyclin-dependent kinase inhibitors P21 and P27, as well as the key apoptosis regulator P53 can induce cell cycle arrest. CX-5461 treatment significantly increased the accumulation of P21, P27, and P53 (Fig. 2*D*). In addition, CX-5461 treatment activated the DNA damage markers p-H2AX and p-CHK1 (Fig. 2, *D* and *E*). Collectively, these results show that CX-5461 effectively inhibited pre-45S rRNA expression, arrested the cell cycle at the G2 phase, triggered a DNA damage response, and promoted apoptosis in NSCLC cells.

### Nucleolar protein DCAF13 regulates pre-45S rRNA expression in NSCLC cells

**Figure 3:**
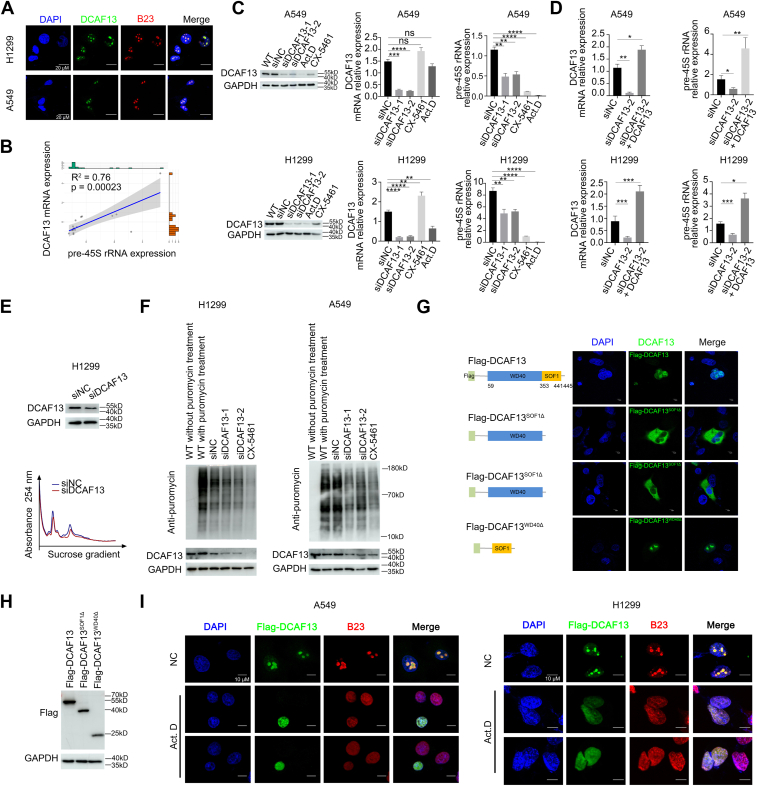
**Silence of DCAF13 decreased the pre-45S rRNA expression, ribosomal biogenesis, and protein synthesis.***A*, immunofluorescent staining results showing that DCAF13 localizes at the nucleolus in H1299 and A549 cells. Cells were cultured and immunostained with anti-DCAF13 antibodies (*green*) and antibodies against the nucleolar marker B23 (*red*). The scale bars represent 20 μm. *B*, scatter plot to assess the correlation between DCAF13 mRNA and pre-45S rRNA in NSCLC tumors (n = 18; R^2^ = 0.76). *C*, H1299 and A549 cells were treated with negative control siRNA, *Dcaf13* siRNA1 or *Dcaf13* siRNA2 for 36 h. After 36 h, cellular DCAF13 level was measured by western blots (*left* panel) and qPCR (*middle* panel). The pre-45S rRNA expression level was measured by qPCR (*right* panel). Cells treated with Act. D (5 μM, 12 h) or CX-5461 (5 μM, 12 h) were used as a positive control. ∗∗*p* < 0.01, ∗∗∗*p* < 0.001, ∗∗∗∗*p* < 0.0001, unpaired *t* test. *D*, cells treated with *Dcaf13* siRNA2 were transfected with DCAF13 plasmids to rescue the expression of DCAF13. The expression level of DCAF13 and pre-45S rRNA was measured by qPCR. *E*, the polysome profiles to monitor ribosome assembly in H1299 cells were analyzed by sucrose density gradient centrifugation. The curve graph showing the polysome profiles of H1299 cells transfected with negative control siRNA or *Dcaf13* siRNA2. *F*, western blot results showing global protein synthesis activity in wild type H1299 and A549 cells or cells treated with negative control siRNA, *Dcaf13* siRNA1, *Dcaf13* siRNA2, or CX-5461 (5 μM) for 36 h. Cells were incubated in DMEM medium containing 2 μM puromycin for 3 h before being harvested for western blots using anti-puromycin antibody. *G*, immunofluorescent staining results showing A549 cells transiently expressing Flag-DCAF13, Flag-DCAF13^SOF1△^, and Flag-DCAF13^WD40△^ were stained with anti-Flag. *H*, western blot results showing the indicated protein Flag-DCAF13, Flag-DCAF13^SOF1△^ and Flag-DCAF13^WD40△^. *I*, H1299 and A549 cells transiently expressing Flag-DCAF13 were treated with 5 μM Act. D for 12 h, and then were fixed and stained with anti-Flag (*green*), anti-B23 (*red*) and DAPI (*blue*). Act. D, actinomycin D; DAPI, 4′,6-diamidino-2-phenylindole; DCAF13, DDB1- and CUL4-associated factor 13; DMEM, Dulbecco's modified Eagle's medium; NSCLC, non–small cell lung cancer; qPCR, quantitative real-time PCR.

Zhang *et al.* revealed that DCAF13 promotes rDNA transcription by mediating CRL4 to direct methyltransferase SUV39H1 for ubiquitination and degradation in preimplantation embryos (24). In addition, Wei *et al.* found that the highly expressed DCAF13 in LUAD is negatively correlated with clinical outcomes in patients with LUAD (32). Therefore, we hypothesized that DCAF13 may act as an rDNA transcription regulator in NSCLC and promotes malignant progression. We found that DCAF13 colocalized with the nucleolar protein B23 and was mainly enriched in the nucleolus of both H1299 and A549 cells (Fig. 3*A*). To test whether DCAF13 expression was positively correlated with pre-45S rRNA expression, we compared DCAF13 and pre-45S rRNA expression levels in paired tumor and adjacent normal tissues and analyzed the correlation between DCAF13 and pre-45S rRNA. The expression level of DCAF13 positively correlated with that of pre-45S rRNA (Fig. 3*B*). Moreover, DCAF13 silencing *via* siRNAs significantly decreased pre-45S rRNA expression in H1299 and A549 cells, while DCAF13 overexpression elevated the level of pre-45S rRNA (Fig. 3, *C* and *D*). The efficiency of DCAF13 depletion and overexpression was determined using western blotting and quantitative real-time PCR.

Given that DCAF13 depletion resulted in decreased pre-45S rRNA expression, we speculated that DCAF13 depletion may affect ribosome biogenesis. Polysome profiling analysis revealed that 60S ribosomal peaks and 40S ribosomal peaks were decreased in siDCAF13-transfected H1299 cells when compared with control cells (Fig. 3*E*). Ribosomal biogenesis is a prerequisite for protein synthesis. To investigate the effects of DCAF13 on protein synthesis, wild-type and DCAF13-depleted H1299 and A549 cells were incubated with puromycin. Puromycin, an analog of transport RNA (tRNA), carries amino acids that are incorporated into newly formed peptide chains. Higher protein synthesis results in greater puromycin incorporation. As shown in Figure 3*F*, similar to CX-5461 treatment, DCAF13 knockdown decreased global protein synthesis. These results indicate that DCAF13 depletion results in a decrease in pre-45S rRNA expression, ribosome biogenesis, and protein synthesis.

DCAF13 is a highly conserved protein containing seven WD40 repeats at its N terminus and an SOF1 domain at its C terminus. To define the domain of DCAF13 that is responsible for the localization of DCAF13 to the nucleolus, we generated FLAG-tagged WT full-length DCAF13 and FLAG-tagged truncated DCAF13 mutants (DCAF13^WD40△^ or DCAF13^SOF1△^) and determined their localization in A549 cells (Fig. 3*H*). We found that, similar to endogenous DCAF13, FLAG-tagged DCAF13 also localized to the nucleolus (Fig. 3*G*). However, FLAG-tagged DCAF13^SOF1△^ failed to localize to the nucleolus or nucleoplasm, whereas FLAG-tagged DCAF13^WD40△^ retained nucleolar association (Fig. 3*G*). These results indicated that the nucleolar signal of DCAF13 is present in its SOF1 domain.

Many rDNA transcription regulators, such as Sirt7, FLNA, and DOR, undergo nucleolar–nucleoplasmic translocation upon treatment with low concentrations of actinomycin D (Act. D), which preferentially inhibits POLR1. We found that upon treatment of A549 and H1299 cells with Act. D, DCAF13 was evenly distributed in the nucleolus and nucleoplasm, rather than being mainly enriched in the nucleolus (Fig. 3*I*), further indicating the involvement of DCAF13 in rDNA transcription. Taken together, these results demonstrate that the nucleolar protein DCAF13 serves as an rDNA transcription regulator in NSCLC, and DCAF13 knockdown reduces pre-45S rRNA expression, ribosome biogenesis, and protein synthesis.

### DCAF13 interacts with TAF1A and is necessary for the assembly of the RNA polymerase I preinitiation complex

**Figure 4:**
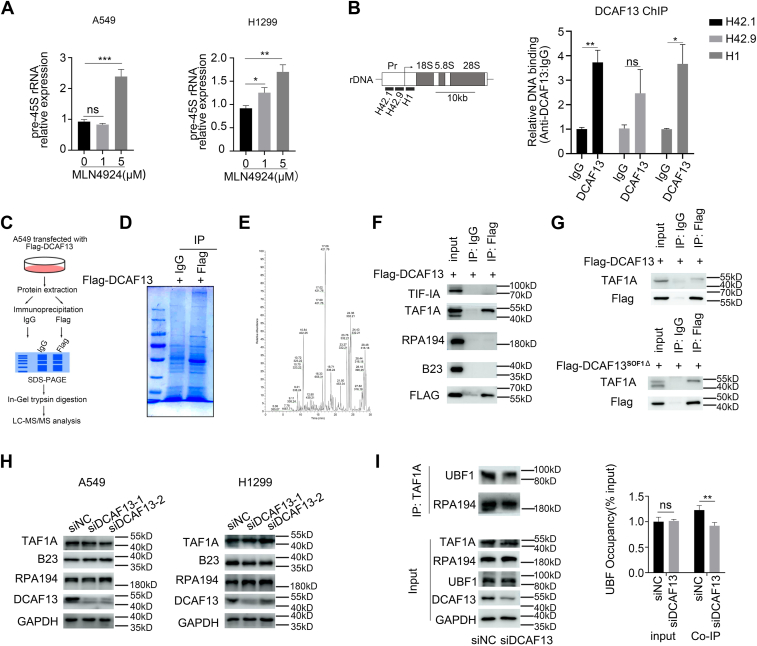
**The interaction between DCAF13 and TAF1A is required for rDNA transcription.***A*, qPCR results showing the changes of pre-45S rRNA expression level in A549 and H1299 cells treated with increased concentrations of MLN4924 for 24 h. *B*, schematic representation of the human rDNA. The positions of primer pairs used in ChIP assays are indicated (*left*). ChIP assay of the enrichment of rDNA in DCAF13 immunoprecipitates (*right*). *C*, a schematic view of mass spectrometry analysis of DCAF13 interacting proteins. *D*, coomassie brilliant blue staining of proteins pulled with IgG antibody and Flag antibody. Flag-DCAF13 plasmid was overexpressed in this experiment. *E*, the base peak of protein sample by mass spectrometry. *F*, coimmunoprecipitation of TIF-IA, TAF1A, RPA194, B23 with Flag-DCAF13 in 293T cells. Flag-DCAF13 was immunoprecipitated with anti-Flag. *G*: Coimmunoprecipitation of TAF1A with Flag-DCAF13 or Flag-DCAF13^SOF1△^ in 293T cells. *H*, western blot results showing the TAF1A, B23 and RPA194 expression level in A549 and H1299 cells treated with negative control siRNA, *Dcaf13* siRNA-1 or *Dcaf13* siRNA-2. *I*, *Dcaf13* siRNA-2 treated cells were subjected to immunoprecipitation by anti-TAF1A, followed by western blot to detect the indicated proteins RPA194 and UBF1. ChIP, chromatin immunoprecipitation; DCAF13, DDB1- and CUL4-associated factor 13; IgG, immunoglobulin G; rDNA, ribosomal DNA; qPCR, quantitative real-time PCR; UBF1, upstream binding factor 1.

Considering that DCAF13 acts as a substrate-recognition protein for the CRL4 E3 ligase, we examined whether the decreased expression of pre-45S rRNA owing to DCAF13 depletion was due to a change in the activity of CRL4 E3 ligase. We found that, in contrast to the decrease in pre-45S rRNA expression induced by DCAF13 silencing, knockdown of ROC1, a component of CRL4 E3 ligase, significantly increased pre-45S rRNA expression (Fig. S1*A*). The small molecule inhibitor MLN4924 selectively blocks the activity of NEDD8 activating enzyme, thereby hindering Cullin neddylation and preventing the activation of Cullin RING E3 ubiquitin ligases. MLN4924 treatment increased rather than decreased pre-45S rRNA expression (Fig. 4*A*), indicating that DCAF13 regulates rDNA transcription independent of CRL4 E3 ligase activity. Regulation of rDNA transcription by DCAF13 suggests a potential association of DCAF13 with rDNA. We performed a chromatin immunoprecipitation (ChIP) assay to check the interaction between DCAF13 and rDNA promoters. The DNA precipitated by DCAF13 antibody was amplified by real time PCR using three primer sets distributed spanning the rDNA promoter (Fig. 4*B*). We found that DCAF13 is enriched in the promoter regions of rDNA (H42.1 and H1), and less presented in the region of rDNA (H42.9). These results indicated DCAF13 could bind to the promoter of rDNA.

To identify the specific target substrate of DCAF13, we performed proteomic analysis of DCAF13-interacting proteins to reveal the potential mechanisms underlying DCAF13 regulation of rDNA transcription. Immunoprecipitation with anti-FLAG antibodies (Abs) followed by mass spectrometry was performed to identify the proteins that specifically interacted with FLAG-DCAF13 (Fig. 4*C*). Coomassie brilliant blue staining displayed the proteins pulled down by immunoglobulin G (IgG) and FLAG Abs (Fig. 4*D*). Several potential DCAF13-interacting proteins were identified, including TAF1A (Fig. 4*E*). TAF1A, a subunit of selective factor 1 (SL1), is a TATA box-binding protein-associated factor that plays a role in the assembly of the RNA polymerase I preinitiation complex (PIC). To further validate the interaction between DCAF13 and TAF1A, we overexpressed FLAG-DCAF13 in 293T cells and performed an immunoprecipitation assay using anti-FLAG Abs or IgG Abs. As shown in Figure 4*F*, FLAG-DCAF13 specifically interacted with TAF1A but not with other components of the RNA polymerase I PIC, such as RPA194 (a subunit of Pol I), TIF-IA, or B23. We also determined the interaction ability of the DCAF13 truncations with TAF1A and found that DCAF13^SOF1△^ interacts with TAF1A (Fig. 4*G*), indicating that WD40 domain are responsible for the interaction between DCAF13 and TAF1A. To determine whether the overexpression of DCAF13^SOF1Δ^ could rescue the pre-45S rRNA expression defects caused by DCAF13 deficiency, we expressed WT DCAF13 and DCAF13^SOF1Δ^ respectively in siDCAF13-transfected H1299 cells and found that overexpression of DCAF13 rather than DCAF13^SOF1Δ^ could rescue the expression of pre-45S rRNA (Fig. S1*B*). We speculate that it might be because DCAF13^SOF1Δ^ loses its nucleolar localization and thus cannot regulate rDNA transcription.

To test if TAF1A is necessary for rDNA transcription in NSCLC cells, we found that similar to DCAF13 depletion, TAF1A knockdown also decreased the level of pre-45S rRNA and global protein synthesis (Fig. S1*C*), indicating that TAF1A is involved in rDNA transcription. To determine whether DCAF13 affect the expression of PIC components, we observed that the protein levels of TAF1A, RPA194, and B23 were not altered in DCAF13-siRNA-treated cells (Fig. 4*H*), indicating that the decrease in rDNA transcription levels induced by DCAF13 knockdown was not due to a reduction in the expression of PIC components. Given that SL1 forms a complex with the upstream binding factor 1(UBF1) at the rDNA promoter region and recruits Pol I to form the transcription machinery, we investigated the possible need of DCAF13 for the association of SL1 with other components of the rDNA transcriptional machinery. As shown in Figure 4*I*, the knockdown of DCAF13 impaired the binding of TAF1A to UBF1. These results suggest that DCAF13 is required for the assembly of rDNA transcription machinery at the rDNA promoter, thereby facilitating transcription initiation.

### DCAF13 promotes NSCLC progression

**Figure 5:**
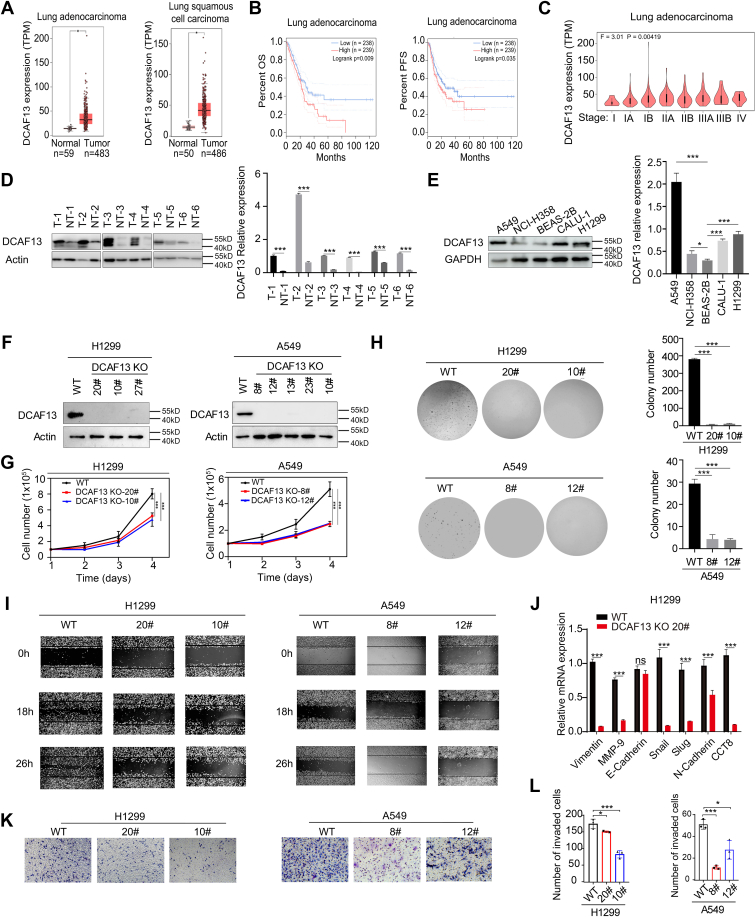
**DCAF13 promotes non–small cell lung cancer progression.***A*, DCAF13 mRNA expression level by UALCAN. *B*, association between DCAF13 mRNA expression and prognosis in TCGA LUAD samples by Kaplan–Meier analysis (log-rank test). *C*, association between DCAF13 mRNA expression and pathologic staging. *D*, western blot results showing the DCAF13 expression level in NSCLC adjacent paraneoplastic tissues (NT) and tumor tissues (T). *E*, expression levels of DCAF13 in lung cancer cell lines (A549, NCI-H358, CALU-1, and H1299) and normal pulmonary bronchial epithelial cell (BEAS-2B) were evaluated by western blot. *F*, deletion of DCAF13 was confirmed at the protein level in different clones of H1299 and A549 cells by immunoblotting. *G*, growth curve assay for D*CAF13* deletion H1299 and A549 cells. Cells (100,000) were plated in six-well culture dishes and cells were counted on days 1, 2, and 3. The data (N = 3 per cell line) are plotted as mean ± SEM. ∗∗∗*p* < 0.001, unpaired *t* test. *H*, DCAF13 deletion inhibits colony formation in H1299 and A549 cells. Data are presented as mean ± SEM for N = 3 per cell line. ∗∗∗*p* < 0.001. *I*, wound healing assay was performed for evaluating the migration ability of DCAF13 deletion H1299 and A549 cells. *J*, the expression of several key cell metastatic signal regulators, vimentin, MMP-9, E-cadherin, slug, snail, N-cadherin, and CCT8 were examined by qPCR. ∗∗∗*p* < 0.001, unpaired *t* test. *K*, Transwell migration assay with the 24-well transwell system and quantitative analysis (original magnification: × 100). *L*, the numbers of migrated cells were counted in five representative high-power fields per transwell plate, ∗∗*p* < 0.01. DCAF13, DDB1- and CUL4-associated factor 13; LUAD, lung adenocarcinoma; NSCLC, non–small cell lung cancer; qPCR, quantitative real-time PCR.

To examine the role of DCAF13 in the progression of NSCLC, we used the UALCAN database to analyze DCAF13 expression and its correlation with prognosis in TCGA LUAD and LUSC samples. As shown in Figure 5*A*, the expression of DCAF13 in LUAD and LUSC tumor tissues was significantly higher than that in normal lung tissues. We further examined the correlation between DCAF13 expression and clinical prognosis in patients with NSCLC and found that LUAD and LUSC patients with high expression of DCAF13 exhibited lower overall survival (Fig. 5B). In addition, DCAF13 expression was positively correlated with clinical stage (Fig. 5C). To investigate whether DCAF13 protein levels were consistent with the results from the public database, we compared the expression of DCAF13 in matched clinical NSCLC tumor tissues and adjacent nontumor tissues. We found that, compared with adjacent tissues, DCAF13 was highly expressed in the NSCLC tumor tissues (Fig. 5*D*). These results suggest that DCAF13 is overexpressed in NSCLC and that high expression is associated with poor prognosis in NSCLC patients.

To investigate the functional significance of DCAF13 in NSCLC *in vitro*, we first examined its expression status in NSCLC cell lines (A549, H1299, NCI-H358, and CALU-1) and normal pulmonary bronchial epithelial cells (BEAS-2B). Three NSCLC cell lines (A549, H1299, and Calu-1) exhibited higher DCAF13 expression than BEAS-2B cells at the mRNA and protein levels (Fig. 5*E*). DCAF13 depletion also decreased the expression of pre-45S rRNA in BEAS-2B cells (Fig. S1*D*), suggesting that DCAF13 regulates rDNA transcription both in BEAS-2B cells and NSCLC cells. Using CRISPR/Cas9 technology, we knocked out *DCAF13* in two NSCLC cell lines and successfully obtained multiple A549 and H1299 cell clones with *DCAF13* deletions (Fig. 5*F*). The cell proliferation assay showed that *DCAF13* knockout significantly decreased the proliferation of A549 and H1299 cells (Fig. 5*G*). Colony formation assay showed that *DCAF13* deletion reduced the number of colonies formed (Fig. 5*H*). To demonstrate that *DCAF13* deletion affected cell migration, wound healing assays was performed and showed that DCAF13 deletion significantly inhibited NSCLC cell migration (Fig. 5*I*). The transwell assays further confirmed that *DCAF13* deletion suppressed NSCLC cell migration (Fig. 5, *K* and *L*). Next, we determined the expression levels of several metastatic phenotype markers in NSCLC cells and found that *DCAF13* deletion downregulated the mesenchymal markers vimentin, MMP-9, N-cadherin, Snail, and Slug, and upregulated the epithelial marker E-cadherin in H1299 cells (Figs. 5*J* and S1*E*). Collectively, these data demonstrate the important role of *DCAF13* in promoting the proliferation and migration of NSCLC cells.

### DCAF13 knockout suppressed NSCLC cell proliferation *in vivo*

**Figure 6:**
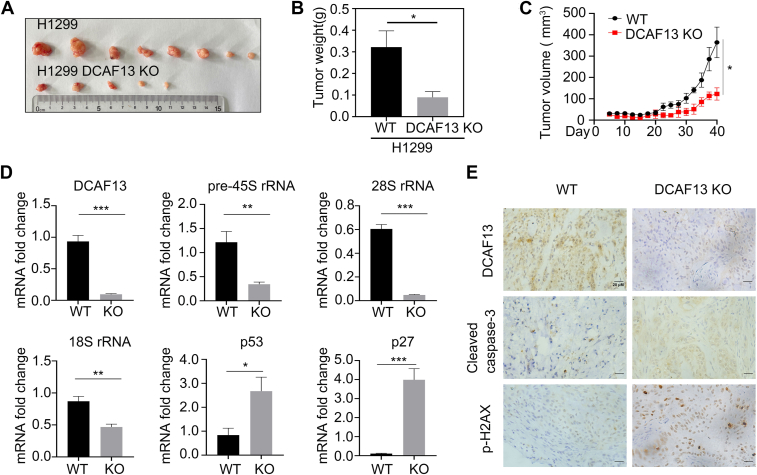
**DCAF13 deletion inhibited the NSCLC cell proliferation *in vivo*.***A*, representative photographs of tumors formed by WT and DCAF13 deletion H1299 cells in nude mice. *B*, tumor weight was determined at the end of the experiments. ∗*p* < 0.05, unpaired *t* test. *C*, the volume of subcutaneous tumors was monitored. ∗*p* < 0.05, unpaired *t* test. *D*, RNA was extracted from the tumors in WT group and DCAF13 deletion group. The expression level of pre-45S rRNA, 28S rRNA, 18S rRNA, p53 and p27 in the tumors was determined by qPCR. Data are mean ± SEM. ∗*p* < 0.05, ∗∗*p* < 0.01, ∗∗∗*p* < 0.001, unpaired *t* test. *E*, immunohistochemical analysis of DCAF13, p-H2AX and cleaved Caspase-3 in WT and DCAF13 deletion tumor tissues. DCAF13, DDB1- and CUL4-associated factor 13; qPCR, quantitative real-time PCR.

To verify the effect of DCAF13 on NSCLC cell proliferation *in vivo*, we subcutaneously implanted WT and DCAF13 knockout H1299 cells into the left and right flanks of nude mice, respectively, and periodically measured the tumor size formed by WT and DCAF13 knockout H1299 cells. We observed that the volume and weight of tumors formed by DCAF13 knockout H1299 cells was smaller than that of the control group (Fig. 6, *A*–*C*), suggesting that DCAF13 knockout inhibited NSCLC cell proliferation *in vivo*. Quantitative PCR results showed that DCAF13 knockout decreased the expression of pre-45S rRNA (Fig. 6*D*). The generated pre-45S rRNA underwent a series of shear processing and modification, and finally formed mature products 5.8S, 18S, and 28S rRNA. Quantitative PCR results also demonstrated that DCAF13 KO decreased the expression of 28S rRNA and 18S rRNA (Fig. 6*D*). These results indicated that DCAF13 deletion downregulated rDNA transcripts. Besides, DCAF13 KO increased the expression of p53 and p27. Immunohistochemical results showed that DCAF13 knockout reduced the expression of cell proliferation marker Ki67, and increased the expression of apoptosis protein cleaved caspase-3, and DNA damage marker p-H2AX (Fig. 6*E*), further confirming that DCAF13 knockout inhibited cell proliferation and promoted apoptosis of H1299 cells *in vivo*.

### rDNA transcription inhibitors target DCAF13 to inhibit NSCLC cell proliferation

**Figure 7:**
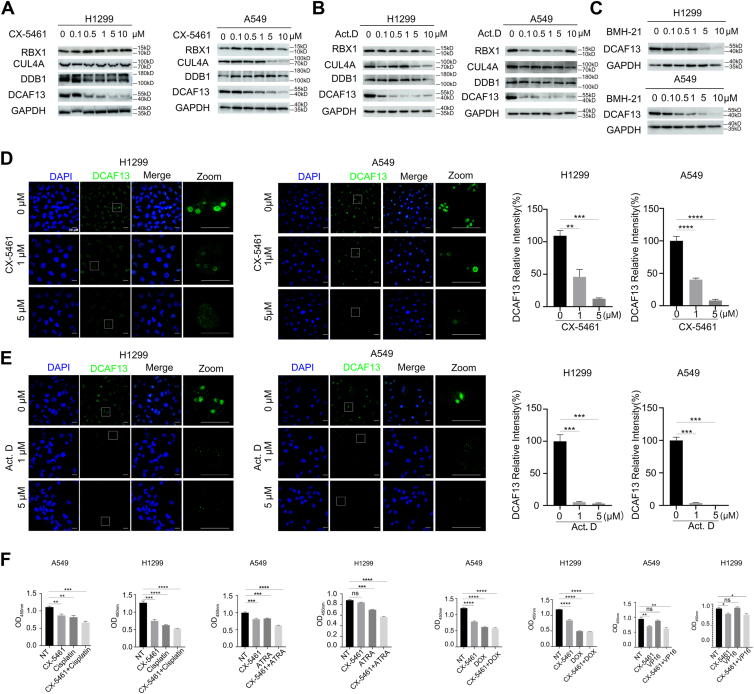
**RNA polymerase I inhibitors decreased the expression of DCAF13.***A*-*C*, western blot results showing the expression level of the components of CRL4 E3 ligase (ROC1, CUL4A, DDB1, or DCAF13) in H1299 and A549 cells treated with increased concentrations of CX-5461 (*A*), Act. D (*B*) or BMH-21(*C*) for 24 h respectively. *D*–*E*, immunofluorescent staining results showing decreased DCAF13 levels at 24 h after treatment with CX-5461(*D*) or Act. D (*E*). ∗∗*p* < 0.01, ∗∗∗*p* < 0.001, ∗∗∗∗*p* < 0.0001, unpaired *t* test. *F*, CX-5461 (0.2 μM) treatment was combined with anticancer agents. A549, H1299 cells were seeded in 96-well plates in triplicate and treated with CX-5461 and the indicated drugs for 48 h, followed by MTT assay. Drug concentrations were cisplatin, 20 μM; doxorubicin (DOX), 25 μM; ATRA, 100 μM; etoposide (VP-16), 50 μM. Act. D, actinomycin D; DCAF13, DDB1- and CUL4-associated factor 13; MTT, 3-(4,5-dimethylthiazol-2-yl)-2,5-diphenyltetrazolium bromide.

Several small molecule inhibitors with high selectivity for Pol I, including CX-5461 and BMH-21, have been developed. These compounds suppress rDNA transcription and ribosome biosynthesis and effectively kill cancer cells. CX-5461 exhibits anticancer activity against various tumors and is currently undergoing clinical trials for hematological and solid malignancies. Preliminary research suggests that CX-5461 blocks rDNA transcription by preventing Pol I from recruiting rDNA promoters and subsequently forming initiation complexes. Interestingly, we found that CX-5461 and BMH-21 at a concentration of 0.5 μM significantly decreased the expression of DCAF13 in A549 and H1299 cells. Moreover, as the concentrations of CX-5461 and BMH-21 increased, the expression of DCAF13 gradually decreased (Fig. 7, *A* and *C*). The rDNA transcription inhibitor Act. D also significantly inhibited DCAF13 expression (Fig. 7*B*). However, it seemed that CX-5461 and Act. D did not affect the expression of the other components of CRL4 E3 ligase, namely, CUL4A, ROC1, and DDB1, at concentrations below 10 μM (Fig. 7, *A* and *B*). Immunofluorescence results showed that both CX-5461 and Act. D treatment decreased the fluorescence signal of DCAF13 (Fig. 7, *D* and *E*). Given that our previous results showed that DCAF13 regulates rDNA transcription, we believe that the rDNA transcription inhibitors suppress rDNA transcription by targeting DCAF13. These results suggest that rDNA transcription inhibitors resulted in a reduction in the expression of DCAF13.

Next, we investigated the chemosensitizing effects of CX-5461 in NSCLC cells. We first demonstrated that anticancer agents such as cisplatin, doxorubicin (DOX), ATRA, and etoposide (VP-16) inhibited the proliferation of NSCLC cell lines A549, H1299 at concentrations of 20, 25, 100, and 50 μM, respectively. Subsequently, we fixed the concentration of CX-5461 at a suboptimal dose (0.2 μM) and then determined whether CX-5461 sensitizes NSCLC cells to these anticancer drugs. In A549 and H1299 cells, CX-5461 showed significant additive effects with two anticancer drugs (cisplatin and ATRA) but with a little additive effects with another anticancer drugs (VP16 and DOX) ((Fig. 7*F*). These results implied that CX-5461 may enhance the effects of other chemotherapeutic drugs on NSCLC cells.

KRAS is one of the most common mutant oncogenes in NSCLC. G12C mutation accounts for approximately 40% of all KRAS mutations (35). It has been reported that the mutated KRAS can activate downstream signaling pathways such as MAPK and PI3K, which significantly promotes ribosome biogenesis (36, 37), such as enhancing the transcriptional efficiency of RNA polymerase I, reshaping the structure and function of the nucleolus, and increasing the expression levels of ribosome proteins, for establishing an efficient ribosome biogenesis system and maintain protein synthesis for cancer cell growth. We determined whether the rDNA transcription inhibitor BMH-21 sensitizes the KRAS G12C-mutated NSCLC cells (H358) to the KRAS (G12C) inhibitor D-1553 (garsorasib) (38). As shown in Fig. S1, *F*–*G*, BMH-21 showed significant additive effects with D-1553. The combined application of D-1553 and BMH-21 increased the expression of the tumor suppressor P53 and its downstream gene BAX involved in apoptosis, suggesting that the combined application of D-1553 and BMH-21 promoted the apoptosis of H358 cells.

## Discussion

**Figure 8:**
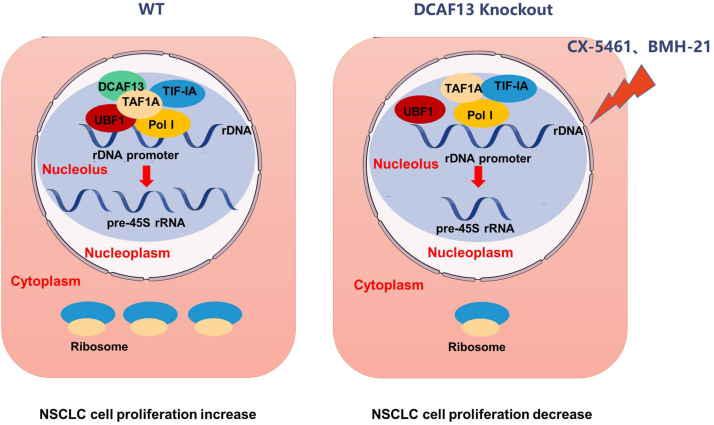
**Schematic diagram of the roles of DCAF13 in non–small cell lung cancer.** Under physiological conditions, DCAF13 localizes at the nucleolus, where it interacts with TAF1A and promotes rDNA transcription. Meanwhile, when DCAF13 was deleted, or the expression of DCAF13 was decreased by CX-5461, Act. D, BMH-21, the formation of the transcription initiation complex was impaired, thereby repressing rDNA transcription, ribosome biogenesis and protein synthesis, which lead to the decreased proliferation of NSCLC cells. Act. D, actinomycin D; DCAF13, DDB1- and CUL4-associated factor 13; NSCLC, non–small cell lung cancer; rDNA, ribosomal DNA.

Abnormal growth and proliferation of cancer cells depend on an increase in protein synthesis and translation, which requires overactivated ribosome biogenesis (10, 11, 12). rDNA transcription, a rate-limiting step in ribosome biogenesis, is tightly regulated to meet the demands for enhanced protein synthesis in cancer. Accumulating evidence shows that an increased level of rDNA transcription is closely correlated with the progression of NSCLC (13, 14). Therefore, identifying novel rDNA transcription regulatory factors and revealing their specific regulatory mechanisms will facilitate the development of therapeutic strategies targeting ribosome production in NSCLC. In the present study, we found that pre-45S rRNA, the rDNA transcription product, was highly expressed in NSCLC tumor tissues compared with that in normal lung tissues. The inhibition of rDNA transcription by CX-5461 significantly decreased NSCLC cell proliferation, both *in vivo* and *in vitro*. Importantly, we identified the nucleolar protein DCAF13, located in the nucleolus of NSCLC cells, which interacts with TAF1A, a component of the rDNA transcription PIC. DCAF13 deficiency inhibited rDNA transcription, ribosome biogenesis, and protein synthesis (Fig. 8). DCAF13 expression is positively associated with pre-45S rRNA expression. In addition, higher DCAF13 expression in NSCLC tumor tissues is a poor prognostic indicator for patients with NSCLC. These results suggest that DCAF13 participates in rDNA transcription to maintain high ribosome production and support NSCLC cell proliferation.

DCAF13 promotes LUAD progression (31, 32). The most important tumor suppressor p53 governs various critical cellular processes, including cell cycle arrest, apoptosis, DNA repair, senescence, autophagy, or metabolism. The stability of p53 is modulated by a variety of post translational modifications, such as ubiquitination and ubiquitin-like modifications. Therefore, identifying the regulatory factors that affect the stability of p53 is of great significance for the development of cancer therapeutic targets. Wei *et al.* (31, 32) found that DCAF13 was highly expressed in LUAD and negatively correlated with the clinical outcomes in LUAD patients, suggesting that the high DCAF13 expression is a poor prognostic indicator in LUAD. Mechanistically, DCAF13 binds with p53, leading to K48-linked ubiquitination and degradation of p53, thereby inhibiting p53 signaling pathway and promoting LUAD progression. This study identified that DCAF13 is a negative regulator of p53, and may be a potential therapeutic target for treating aggressive cancer types. Moreover, p53 could be activated by various cellular stress signals, including nucleolar stress (39). Nucleolar stress was caused by perturbations of ribosome biogenesis, such as the inhibition of rRNA processing, synthesis, and ribosome assembly. Under this stress condition, several ribosomal proteins translocate from the nucleolus to the nucleoplasm, where they interact with MDM2 and inhibit MDM2-mediated ubiquitination and degradation toward p53, consequently resulting in the activation of p53. In the present study, we found that DCAF13 participated in rDNA transcription in NSCLC. DCAF13 depletion impaired ribosome biogenesis, which triggers nucleolar stress and leads to the accumulation of p53 protein. Thus, DCAF13 could affect the level of p53 protein by directly binding to p53, regulating its ubiquitination and degradation. Meanwhile, DCAF13 may also regulate the p53 pathway through the nucleolar stress.

The effective initiation of rDNA transcription requires the formation of a transcription PIC at the rDNA promoter (9). The PIC includes Pol I, UBF, SL1, TIF-1A, and other transcription factors. A key step in the PIC assembly is the binding of the UBF dimer to the rDNA promoter. This is followed by the recruitment of SL1 to form the UBF/SL1 complex. Finally, SL1 recruits Pol I to the rDNA promoter *via* interaction with TIF-1A, initiating the transcription of rDNA genes. Many classic signaling pathways control cell growth and proliferation by acting on these protein-protein and protein rDNA interactions. For example, the oncogene *c-Myc* interacts directly with SL1 to increase rDNA transcription and promote cell proliferation (40). P53 interacts directly with SL1, preventing SL1 from interacting with the UBF and inhibiting rDNA transcription (19). In our study, we found that DCAF13 specifically interacts with TAF1A, a member of the SL1 complex, but not with TIF-1A or RPA194 (a component of Pol I). Silencing DCAF13 hampered the interaction between TAF1A and UBF1, indicating that DCAF13 may serve as a molecular scaffold to promote the assembly of RNA polymerase I PIC.

The nucleolus is the subcellular structure of rRNA synthesis and ribosome subunit assembly, which determines cell protein synthesis and cell homeostasis. The nucleolus is typically a three-layer compartmented structure where many proteins are localized and involved in the construction of the liquid phase stratification of the fiber center, dense fiber component, and granular component. Current studies have shown that the nucleolus mainly maintains its normal multilevel structure and function through liquid-liquid phase separation of various biological macromolecules. Nucleolar phase separation is a crucial molecular event required for ribosome biogenesis, protein translation, and nucleolar stress response (41). Zhou *et al.* reported that DCAF13 promoted NPM1 phase separation, enhanced pre-rRNA enrichment, and promoted 18S rRNA maturation by recruiting endonuclease UTP23 during T cell proliferation (26). Therefore, DCAF13 may act as a phase separation regulator of rDNA transcription. The nucleolar, a nonmembranous organelle, is not only responsible for ribosome biogenesis but also serves as a cellular stress sensor, playing a central role in the cellular stress response network (42). Nucleolar stress is caused by a variety of cellular stress factors, such as chemical stress, heat shock, oxidative stress, hypoxia, and UV radiation, causing abnormalities in nucleolar structure and function, rRNA synthesis and processing, and ribosome assembly. These disruptions can activate P53 or other stress pathways, leading to apoptosis. In this study, DCAF13 shifted from the nucleolus to the nucleoplasm following Act. D treatment, implying that DCAF13 may also play a role in nucleolar stress.

In summary, our study revealed a new mechanism of DCAF13 in regulating rDNA transcription to promote oncogenic phenotypes of NSCLC. Our findings underscore the importance of DCAF13-mediated regulation of the RNA polymerase I transcriptional machinery and open new avenues for targeted cancer therapies aimed at disrupting this pathway to inhibit tumor growth and metastasis.

## Experimental procedures

### Cell culture and reagents

Human NSCLC cell lines (A549, H1299, Calu-1, and NCI-H358), normal pulmonary bronchial epithelial cells (BEAS-2B), and human embryonic kidney cells (293T) were purchased from the American Type Culture Collection. A549, H1299, BEAS-2B, and 293T cells were cultured in Dulbecco's modified Eagle's medium (DMEM) (Gibco). Calu-1 and NCI-H358 cells were cultured in RPMI 1640 medium (Gibco). Both DMEM and RPMI 1640 were supplemented with 10% fetal bovine serum (FBS; Gibco) and 1% penicillin-streptomycin solution (Gibco). All cells were incubated at 37 °C in a humidified incubator with 5% CO_2_. All anti-cancer drugs, including Cisplatin (HY-17394), VP-16(HY-13629), ATRA (HY-14649), and DOX (HY-15142), were purchased from MedChemExpress (MCE).

### RNA isolation and quantitative PCR

The cells in the six-well plates were lyzed with TriZol (RN0101; Aidlab), and total RNA was isolated according to the manufacturer's protocol. RNA was reverse-transcribed into complementary DNA by using a PrimeScript RT Master Mix kit (RR036A; TaKaRa). Quantitative real-time PCR (qPCR) was performed using SYBR GREEN PCR Master Mix (RR820A; TaKaRa) on an ABI7500 Real-Time PCR System (Applied Biosystems). For gene expression analysis, β-actin was used as internal controls. The following primers used to amplify target genes were in Table S1.

### Mouse and xenograft models

Briefly, 6-8-week-old BALB/c nude mice (GemPharmatech) were kept in an specific pathogen free house with 14 h of light and 10 h of darkness a day and provided food and water regularly. All animal experiments follow the relevant regulations of national experimental animal welfare ethics, and has been approved by the Laboratory Animal Ethics Committee of JXMC (Registration No. JUMC2022-046). To evaluate the effect of CX-5461 on the proliferation of NSCLC cells *in vivo*, 5 × 10^6^ cells were injected subcutaneously into both sides of the back of mice. Mice were randomly divided into control and CX-5461 treatment groups with six mice in each group. After the cells had formed subcutaneous tumors, the mice in the two groups were fed water and CX-5461 (50 mg/kg) every 2 days *via* gavage. Tumor volume was measured and calculated using the following formula: volume (mm^3^) = 1/2 × length × width^2^. When the tumor diameter reached 15 mm, primary tumor masses were excised, weighed, and stored at −80 °C for subsequent quantitative PCR.

### siRNA-mediated gene silencing

Cells were seeded in six-well plates at a density of 2.5 × 10^5^ cells per well. When the cell density was about 60%, cells were transfected with siRNAs by using transfection reagent Lipofectamine RNAiMAX (13778150; Invitrogen). Briefly, 100 μl Opti-MEM and 10 μl transfection reagents were incubated in one 1.5 ml centrifuge tube for 5 min, and 100 μl Opti-MEM and 6 μl siRNA (100 nmol) were incubated in another 1.5 ml centrifuge tube for 5 min, respectively. After that, the liquid in the two tubes was mixed to form liposome complex, which was incubated for 15 min at room temperature. The mixture was added to cells plated in 1 ml of DMEM medium supplemented with FBS and placed in an incubator at 37 °C for 36 h. The siRNA sequences were in Table S2.

### Immunohistochemistry

Mouse tumor tissues were fixed, embedded in paraffin, sliced into 5 μm thick sections and deparaffinized. The deparaffinized sections were incubated in 0.3% hydrogen peroxide for 10 min to inactivate endogenous peroxidase. The slices were soaked in 10 mm sodium citrate (pH = 6.0) in a pressure cooker, heated for 2 min at full pressure for antigen repair, and then cooled at room temperature. The slices were blocked with PBST (1 × PBS + 0.1% Tween-20) containing 10% goat serum for 30 min, incubated with primary Abs in a wet box at 4 °C overnight, and incubated with horseradish peroxidase-conjugated secondary Abs for 30 min. The sections were counterstained utilizing a Vectastain ABC kit and a 3,3′-diaminobenzidine peroxidase substrate kit (Vector Laboratories).The primary Abs against Ki-67, p-H2AX, cleaved caspase-3, and Brdu were in Table S3.

### Western blot analysis

The extracted proteins were quantified by bicinchoninic acid (BCA) protein assay kit (P0012; Beyotime). Twenty micrograms of proteins was mixed with 6 × loading buffer and denaturated at 95 °C for 5 min. The denatured proteins were separated by sodium dodecyl sulfate-polyacrylamide gel electrophoresis. Polyvinylidene fluoride (PVDF) membranes (Millipore Corp) were activated in methanol for 30 s. The proteins in gel were transferred to PVDF membranes at 300 mA for 1 h, which was blocked by TBST (150 mM NaCl, 10 mM Tris–HCl, pH 7.5, and 0.1% Tween 20) containing 5% (w/v) bovine serum albumin (F-0332; haoxinbio.tech). The PVDF membranes were washed with TBST buffer for three times, then incubated with the corresponding primary Abs at 4 °C overnight, and incubated with horseradish peroxidase-conjugated anti-rabbit secondary Abs for 1 h at room temperature. Specific bands were analyzed using a chemiluminescence imaging system (Amersham Imager 680; GE Healthcare Technologies Inc). The blots were quantitatively analyzed by ImageJ software. The Abs used in western blot are in Table S3.

### Immunofluorescence staining

Immunofluorescence staining was performed as described previously (28). Briefly, H1299 or A549 cells were seeded on the glass coverslips transfected with plasmids (FLAG-DCAF13, FLAG-DCAF13^SOF1△^, or FLAG-DCAF13^WD40△^) for 36 h, or treated with drugs (CX-5461, Act. D) at indicated concentrations for 12 h. Cells were then washed with PBS and fixed with 4% paraformaldehyde for 30 min. Then the cells were washed with PBS three times, and blocked by PBS containing 0.3% Triton X-100 and 5% bovine serum albumin for 1 h. Cells were then incubated with primary Abs at 4 °C overnight and secondary Abs coupled to Alexa Fluor 594 or 488 in darkness for 1 h at room temperature. The cells were then washed with PBS three times and incubated with 4′,6-diamidino-2-phenylindole (DAPI) (HY-D0814; MCE) for nuclear staining for 5 min. The glass coverslips were sealed with 50% glycerin. Cell images were acquired using a laser scanning confocal microscope (FV3000; Olympus) with a 4 to 100 objective lens. The primary and secondary Abs used in the immunostaining were in Table S3.

### Cell proliferation and viability assay

For cell growth curve analysis, cells were seeded in a 6-wells plate (Corning) in triplicate at a density of 1 × 10^5^ or 1.5 × 10^5^ cells/well and allowed to adhere overnight before exposure to CX-5461. The amount of cells in each well was counted using a hemocytometer 24, 48, and 72 h after seeding.

For the cell viability assay, cells were seeded in a 96-wells plate in quadruplicate at a density of 3 × 10^3^ cells per well. After the cells adhered, cells were treated with various drugs, either alone or in combination. After 48 h, the medium was replaced with DMEM medium supplemented with 10% FBS and 10% cell-counting kit 8 (HY-K0301; haoxinbio.tech). After culturing for between 1 h at 37 °C, the absorbance was measured at 450 nm using a microplate reader (SynergyHTX; BioTek Instruments, Inc).

### Colony formation assay

PBS containing 1% bottom agar (Sigma-Aldrich) was mixed with equal volume of DMEM supplemented with 10% FBS. The six-well plates were placed with 1.5 ml of the mixture per well as the base layer, which was solidified by cooling. Cell suspension (2 × 10³ cells per well) was mixed with PBS containing 0.7% bottom agar and transferred to the base layer to form the top layer. As the top layer was solidified, 2 ml DMEM supplemented with 10% FBS was added to the top layer. After 3 weeks, the supernant was removed, and the colonies were stained with 0.1% crystal violet (Sigma-Aldrich). ImageJ software 1.8.0 (National Institutes of Health) was used for analysis.

### Scratch and transwell assays

For the scratch assays, cells were seeded in a 6-wells plate at a density of 5 × 10^5^ cells/well in DMEM containing 10% FBS. After adherence, scratches were made using a 10 μl pipette tip, wells were washed with PBS to remove debris, and cells were cultured in FBS-free DMEM. Plates were then imaged at 0 h (baseline) and 24 h using an inverted phase-contrast microscope (CKX53; Olympus).

Transwell assays were performed in 24-wells plate using transwell chambers, which were composed of upper chambers and lower chambers separated by polycarbonate membranes (Corning). Subsequently, 500 μl DMEM containing 10 % FBS was added to the lower chamber of the 24-well plates, and 100 μl of cell suspension (1 × 10^4^ cells) was added to the upper chamber. Cells were cultured overnight at 37 °C with 5% CO_2_ for 24 h. Cells were then washed three times with PBS, fixed with methanol for 30 min, stained with 0.1% crystal violet for 30 min. The unmigrated cells were gently wiped in the upper compartment with a cotton swab. Migrated cells were imaged using an inverted phase-contrast microscope (CKX53; Olympus) and quantified using ImageJ software (National Institutes of Health).

### Flow cytometric analyses of cell cycle

A total of 1 × 10^6^ cells were washed three times with precooled PBS and fixed with 2 ml 70% ethanol at 4 °C overnight. After being centrifuged and washed twice with PBS, cells were resuspended in 500 μl of propidium iodide staining solution (BD Biosciences) and then incubated at 37 °C for 30 min in the dark. Subsequently, cells were assayed *via* flow cytometry (BD FACSVerse; BD Biosciences). The percentages of cells at the G1, S, and G2 phases were analyzed using ModFit software (Verity Software House).

### Coimmunoprecipitation assay

Cells were lysed on ice for 10 min in cell lysis buffer for western blot and immunoprecipitation (P0013; Beyotime). After centrifugation, part of the supernatant was used as the input, while the rest was used for coimmunoprecipitation. Briefly, 50 μl of Protein A/G magnetic beads (HY-K0202; MCE) were washed three times with 400 μl of binding/washing buffer (PBS containing 0.5% Tween-20). The primary Abs diluted in binding/washing buffer (final concentration of the primary Abs: 30 μg/ml) were mixed with Protein A/G magnetic beads and incubated with shaking at 4 °C for 4 h, followed with four washes with washing buffer. Then the cell lysates were transferred into Ab–magnetic beads complexes and incubated overnight at 4 °C. Immunocomplexes were then washed six times with binding/washing buffer, resuspended in 1 × loading buffer, boiled at 95 °C for 5 min, and assayed *via* western blotting.

### Polysome profile and protein synthesis assays

For polysome profile assays, cells were cultured in 10-cm dishes and transfected with nontargeting (siNC) or *Dcaf13* siRNAs for 48 h. Cells were washed twice with pre-cold PBS buffer, scraped, and collected following centrifugation at 12,000 rpm for 5 min at 4 °C. The collected cell pellets were transported on dry ice to Hangzhou NeoRibo Biotechnology Co, Ltd (https://www.neoribo.com/) for polysome profile analysis.

For the puromycin incorporation assay, cells were seeded into 6-well plates and treated with DCAF13 siRNAs for 48 h. Before being lysed, cells were treated with 2 μM puromycin (MCE, HY-B1743A) for 1 h. Nascent protein was detected with anti-puromycin Ab using western blotting.

### ChIP assay

ChIP assay was performed in accordance with the instructions of the ChIP kit (26157; Thermo Fisher Scientific). Briefly, 4 × 10^6^ H1299 cells were cross-linked using 1% formaldehyde for 10 min, then lysed with membrane extraction buffer containing protease inhibitors for 10 min on ice, digested with MNase at 37 °C for 15 min and followed by sonication on ice with three 20-s pulses at 3 W power. The cross-linked, sonicated chromatin was incubated with 5 μg of IgG Abs or DCAF13 Abs and rotated at 4 °C overnight. The overnight mixture was incubated with Protein A/G magnetic beads at 4 °C for 2 h, followed with four washes with washing buffer. After extensive washes, immunocomplexes were eluted by elution buffer at 65 °C for 30 min and treated with proteinase K for reversing crosslinking. Bound DNA in the precipitates was extracted, purified, and subjected to real-time PCR analysis using primers corresponding to different regions of the rDNA promoter. The following primers used to amplify target regions of the rDNA promoter were in Table S1.

### CRISPR/Cas9 technology

CRISPR/Cas9 technology was used to delete DCAF13 in H1299 and A549 cells as described previously (28). Briefly, 293T cells were seeded in 6-well plates at a density of 1 × 10^5^ cells/well. To construct the lentivirus, 293T cells were cotransfected with the recombinant lentiviral vector LentiCRISPRv2 (400 ng) and the packaging plasmids pMD2.G (200 ng) and psPAX2 (200 ng) using poly-jet technology (SignaGen). After 4 h, the virus-containing cell supernatant was filtered using a 0.45-μm hydrophilic PVDF membrane (Millex-HV, Millipore) for removing cell debris. Following this, cells were infected with the filtered virus using polybrene (final concentration 8 μg/ml). After 24 h of transfection, 1 to 2 μg/ml of puromycin (Gibco) was continuously added to the cells for 3 days to sort the transfected cells. Monoclonal cells were sorted into 96-well plates *via* flow cytometry, and DCAF13 knockout efficiency was evaluated *via* western blotting. The guide RNA sequences used for targeting human DCAF13 were: human DCAF13–1: 5′- AGCGGGACAGCAGTGAGCCC-3′; human DCAF13–2: 5′-GATGTGGATTACTCTCCCAC-3′.

### NSCLC tumor samples

Fresh lung tumor tissues and adjacent nonneoplastic tissues were collected from the Jiaxing University First Affiliated Hospital (Jiaxing). The studies in this work abide by the Declaration of Helsinki principles. Informed consent was obtained from all the patients or their legal representatives. This study was approved by the Institutional Review Board and Ethics Committee of the First Affiliated Hospital of Jiaxing University (Registration No. 2023-KY-048).

### Co-immunoprecipitation/MS

DCAF13 was overexpressed by transfecting FLAG-DCAF13 plasmids into A549 cells. When the cell density reached 90%, cells were lysed in western blot and IP lysis buffer (Beyotime Biotechnology). Fifty microliters of Protein A/G magnetic beads (MCE) were washed four times with 400 μl of binding/washing buffer (1 × PBS + 0.5% Tween-20). Following this, the primary Abs against FLAG were incubated with magnetic beads for 4 h at 4 °C. Cell lysates were then transferred to the Ab–magnetic bead complexes and incubated overnight at 4 °C. Immunocomplexes were washed 10 times with a binding/washing buffer, eluted with loading buffer, and heated at 95 °C for 5 min. Immunocomplexes were separated *via* SDS-PAGE and stained with Coomassie brilliant blue. The obtained samples were transported on dry ice to APT Biotechnology for protein identification using liquid chromatography tandem mass spectrometry.

### Statistical analysis

All experiments were conducted using at least three independent biological replicates. All statistical data are presented as mean ± SEM. Statistical analyses were performed using GraphPad Prism software. Statistical significance was determined using an unpaired *t* test. The log-rank test was applied to the Kaplan–Meier analysis, with *p* < 0.05 considered statistically significant. Levels of significance are represented by asterisks as follows: ∗*p* < 0.05, ∗∗*p* < 0.01, and ∗∗∗*p* < 0.001.

## Data availability

The data generated and analyzed during the current study are available from the corresponding author upon reasonable request.

## Supporting information

This article contains supporting information.

## Conflict of interest

The authors declare that they have no conflicts of interest with the contents of this article.

## Figures and Tables

**Supplymentary Figure 1:**