# Supplementary material for: Nucleolar protein DCAF13 promotes non–small cell lung cancer cell proliferation via facilitating rDNA transcription and ribosome biogenesis
Source: J Biol Chem. 2025 Sep 1;301(10):110656. doi: 10.1016/j.jbc.2025.110656 (PMC12514575; doi:10.1016/j.jbc.2025.110656)
Supplement: Supporting Information [file mmc1.docx]

**Nucleolar protein DCAF13 promotes non-small cell lung cancer cell proliferation via facilitating rDNA transcription and ribosome biogenesis**

Xiao-Min Wang^1*^, Meng-Dan Lv^1^, Zhong-Jie Wu^2^, Xiao-Hui Wu^1,3^, Gu-Yuan Sun^1^, Zi-Hao Wu^1^, Xin-Kai Zou^2^, Yu-Tao Wu^1^, Le-Tian Liu^1^, Le-Yi Yin^1^, Yi Zhang^1^, En-Hui Yang^4^, Yong-Xia Zheng^1^, Long Xu^1^, Guo-Xin Hou^2^, Yu Wang^1^, Ya-Ling Zhang^1^, Zi-Wei Hu^1^, Sheng-Bing Liu^1^, Lei Ao^1^, Chun-Wei Xu^5^, Michal Heger^6^, Shu-Qun Cheng^7*^, Wei-Wei Pan^1*^

*^1^* *Department of Basic Medicine, College of Medicine, Jiaxing University, 118 Jiahang Road, Jiaxing 314001, Zhejiang, P. R. China*

*^2^ Department of Cardiothoracic Surgery, Affiliated Hospital of Jiaxing University, Jiaxing314000, Zhejiang, P. R. China*

*^3^ College of Life Sciences and Medicine, Zhejiang Sci-Tech University, Hangzhou 310000, Zhejiang, P. R. China*

*^4^ Alberta Institute, Wenzhou Medical University, Wenzhou 325000, Zhejiang, P. R. China*

*^5^ Institute of Basic Medicine and Cancer (IBMC), Chinese Academy of Sciences, No. 1 Banshan East Street, Gongshu District, Hangzhou 310022, Zhejiang, P. R. China*

*^6^Jiaxing Key Laboratory for Photonanomedicine and Experimental Therapeutics, Department of Pharmaceutics, College of Medicine, Jiaxing University, Jiaxing 314001, Zhejiang, P. R. China*

*^7^Department of Hepatic Surgery VI, Eastern Hepatobiliary Surgery Hospital, Second Military Medical University, 225 Changhai Road, Shanghai 200438, P. R. China*

*Corresponding authors:

Wei-Wei Pan, College of Medicine, Jiaxing University, Jiaxing 314001, P. R. China. Tel: +86-573-85624294; E-mail: wwpan@mail.zjxu.edu.cn

Shu-Qun Cheng, Eastern Hepatobiliary Surgery Hospital, Second Military Medical University, No. 225 Changhai Road, Shanghai 200433, P. R. China. Tel: +86-21-81875251; E-mail: [chengshuqun@aliyun.com](mailto:chengshuqun@aliyun.com)

Xiao-Min Wang, College of Medicine, Jiaxing University, Jiaxing 314001, P. R. China. Tel: +86-573-85624294; E-mail: 11318005@zju.edu.cn

**Running title:** DCAF13 drives NSCLC growth via rDNA transcription regulation

**Supplemental figure legends**

Supplementary Figure. S1A-G

Figure.S1A: western blot results showing the RNAi efficiency of *ROC1* in A549 and H1299 cells with transfection of negative control siRNA or si*ROC1* for 36 h. qPCR results showing the pre-45S rRNA expression level in A549 and H1299 cells treated with control siRNA or si*ROC1.*

Figure.S1B: qPCR results showing the pre-45S rRNA expression level in H1299 cells treated with control siRNA, si*DCAF13*-2, or si*DCAF13*-2 treated H1299 cells tansfected with DCAF13/DCAF13^SOF1△^ plasmids.

Figure.S1C: Western blot results showing global protein synthesis activity in H1299 cells treated with negative control siRNA, *Taf1a* siRNA1, *or Taf1a* siRNA2 for 36 h (left). Cells were incubated in DMEM medium containing 2 μM puromycin for 3 h before being harvested for western blots using anti-puromycin antibody. qPCR results showing the pre-45S rRNA expression level in H1299 cells treated with negative control siRNA, *Taf1a* siRNA1, *or Taf1a* siRNA2 for 36 h (right).

Figure.S1D: qPCR results showing the DCAF13 expression level in BEAS-2B cells treated with control siRNA or si*DCAF13*-2 (left). qPCR results showing the pre-45S rRNA expression level in BEAS-2B cells treated with control siRNA , si*DCAF13*-2 (middle) or different concentrations of CX-5461 (right).

Figure.S1E: Western blot results showing the expression of EMT markers snail and N-Cadherin in H1299 cells treated with negative control siRNA, si*DCAF13*-1, or si*DCAF13*-2 for 36 h.

Figure.S1F: CCK-8 assay showing the cell viability of H358 cells at different concentrations of BMH-21(left), D-1553(right), and treated with BMH-21(1 μM) or D-1553(150 μM) alone or in combination.

Figure.S1G: Western blot results showing the expression of p53 and BAX in H358 cells treated with BMH-21(1 μM) or D-1553(150 μM) alone or in combination for 24 h.

**Table S1. Primers sequences**

| Name | | Sequence |
| --- | --- | --- |
| *β*-actin | 5'-TTGCGTTACACCCTTTCTTG-3'(sense)  5'-CACCTTCACCGTTCCAGTTT-3'(antisense); | |
| pre-45S rRNA | 5'-CCGTCCGTCCGTCGTCCTCCTCGC-3'(sense)  5'-TGTACCGGCCGTGCGTACTTAGAC-3'(antisense); | |
| DCAF13 | 5'-CAGAGAAGCTGGCTACTGTCC-3'(sense)  5'-AGAAGTCCCACAAAAGCGAGT-3'(antisense); | |
| p27 | 5'-AGAAGCACTGCCGGGATATG-3' (sense),  5'-ACCTCCTGCCACTCGTATCT-3'(antisense); | |
| P53 | 5'-ACAACGTTCTGTCCCCCTTG-3'(sense)  5'-CTGGCATTCTGGGAGCTTCA-3'(antisense); | |
| H42.1 | 5'-GCTTCTCGACTCACGGTTTC-3'(sense)  5'-CCGAGAGCACGATCTCAAA-3'(antisense); | |
| H42.9 | 5'-CCCGGGGGAGGTATATCTTT-3'(sense)  5'-CCAACCTCTCCGACGACA-3'(antisense); | |
| H1 | 5'-GGCGGTTTGAGTGAGACGAGA-3'(sense)  5'-ACGTGCGCTCACCGAGAGCAG-3' (anti-sense); | |

**Table S2. siRNA sequences**

| Name | Sequence |
| --- | --- |
| Negative Control | 5'-UUCUCCGAACGUGUCACGUTT-3' |
| siDCAF13-1 | 5'-GUGCUUACAUCACGAGAAAUTT-3'; |
| siDCAF13-2 | 5'-UGUUCAUCCCAAAUGUCUATT -3' |
| siROC1 | 5'-GACUUUCCCUGCUGUUACCUAATT-3'. |
| siTAF1A-1 | 5'- AUUGUAAAUCCAAGACUCUTT-3' |
| siTAF1A-2 | 5'- UGUAAAAGCCCUUUAUAGGCC-3' |

**Table S3.** **Antibodies used in the experiments**

| Antibodies | Source | Cat # |
| --- | --- | --- |
| DCAF13 | Abcam | ab195121 |
| Ki67 | Cell Signaling Technology | #9449 |
| cleaved caspase-3 | Cell Signaling Technology | #9661 |
| Brdu | Cell Signaling Technology | #5292 |
| DDDDK Flag Tag | Abcam | ab205606 |
| B23 | Abcam | ab10530 |
| GAPDH | Abcam | ab8245 |
| Anti-puromycin | Abcam | ab315887 |
| p21 | Cell Signaling Technology | #2947 |
| p27 | Cell Signaling Technology | #3686 |
| TIF-IA | SANTA CRUZ | sc-390464 |
| TAF1A | SANTA CRUZ | sc-393600 |
| RPA194 | SANTA CRUZ | sc-48385 |
| UBF1 | proteintech | 20660-1-AP |
| CUL4A | Cell Signaling Technology | #2699 |
| DDB-1(D4C8) | Cell Signaling Technology | #6998 |
| p-H2AX (Ser139) | Cell Signaling Technology | #80312 |
| p-PDK1 (Ser241) | Cell Signaling Technology | #3061 |
| Anti-rabbit IgG, HRP-linked antibody | Cell Signaling Technology | 7074S |
| Anti-mouse IgG, HRP-linked antibody | Cell Signaling Technology | 7076S |
| Goat Anti-Rabbit IgG  H&L (Alexa Fluor® 488) | Abcam | ab150077 |
| Goat Anti-Mouse IgG  H&L (Alexa Fluor® 594) | Abcam | ab150116 |
